# Impacts of obstructive sleep apnea on collateral circulation in stroke patients with proximal middle cerebral artery stenosis or occlusion

**DOI:** 10.1007/s10072-025-08386-2

**Published:** 2025-08-06

**Authors:** Yuqing Mei, Yanwei Guo, Min Zhang, Wenwei Yun, Gelin Xu

**Affiliations:** 1 Department of Neurology, Affiliated Jingling Hospital, Nanjing Medical University, Nanjing, 210002 Jiangsu China; 2 Depatment of Neurology, Second People’s Hospital of Changzhou, The Third Affiliated Hospital of Nanjing Medical University, Changzhou, China; 3 Department of Neurology, Shenzhen Second People’s Hospital, Inst Translat Med, First Affiliated Hospital of Shenzhen University, Shenzhen, China

**Keywords:** Obstructive sleep apnoea, Collateral circulation, Acute cerebral infarction, Collateral score, Middle cerebral artery

## Abstract

**Background:**

Obstructive sleep apnoea (OSA) is a common sleep-related breathing disorder that can negatively affect the prognosis of cardiovascular events. However, OSA may facilitate the development of collateral circulation via hypoxic preconditioning. This study aimed to investigate the relationship between OSA and intracranial collateral circulation among stroke patients with proximal middle cerebral artery (MCA) stenosis or occlusion.

**Methods:**

Patients with symptomatic unilateral proximal MCA stenosis or occlusion were enrolled. All patients underwent polygraph and computed tomographic angiography within 72 h of stroke onset. OSA was defined as an apnoea-hypopnoea index (AHI) of ≥ 15 events per hour. The collateral circulation of the MCA was visually assessed via the Tan scoring system and ranged from 0 (no collaterals) to 3 (extensive collaterals).

**Results:**

Among the 78 enrolled patients, 74% were male. The mean age of the participants was 61 ± 12 years. OSA was identified in 40 patients (51.3%). Compared with non-OSA patients, OSA patients presented better intracranial collateral circulation (collateral score ≥ 2, 87.5% vs. 65.8%, *P* = 0.023). Additionally, patients with good intracranial collateral circulation (collateral score ≥ 2) had a greater AHI than patients with poor intracranial collateral circulation (25.4 ± 17.1 vs. 14.9 ± 13.2, *P* = 0.02). Spearman’s correlation analysis revealed a positive correlation between the AHI and collateral score (*r* = 0.866, *P* < 0.01).

**Conclusion:**

OSA may facilitate the development of intracranial collateral circulation in patients with proximal MCA stenosis or occlusion and thus exert protective effects among patients with acute cerebral infarction.

## Introduction

Obstructive sleep apnoea (OSA) is a sleep-related respiratory disorder that is characterized by recurrent episodes of complete or partial upper airway collapse during sleep [1]. OSA causes fragmented sleep and frequent nocturnal hypoxia. Furthermore, OSA is highly prevalent among patients with cardiovascular events, affecting up to 60–70% of this population [2–6]. Emerging evidence suggests that OSA may be associated with adverse functional outcomes and an increased risk of mortality among patients suffering from ischaemic stroke [7–10]. These associations have been attributed to several pathophysiological consequences of OSA, including chronic intermittent hypoxia, oxidative stress, inflammatory responses, increased pleural pressure, and increased sympathetic activity [11].

There is an increasing amount of evidence suggesting that OSA exerts protective effects on collateral formation in the heart. However, this influence on intracranial collateral circulation remains underexplored [12–15]. Cerebral collateral circulation refers to the redirection of blood flow through alternative vessels to ischaemic regions. This redirection occurs when the primary cerebral artery is significantly narrowed or occluded, thus providing varying degrees of perfusion compensation to the affected tissue [16]. Although current research predominantly focuses on the impact of OSA on cognitive function, mood disorders, and sleep quality, little attention has been given to its potential effects on collateral circulation in patients with cerebral infarction [17–19]. Therefore, investigating the influence of OSA on collateral circulation in patients with intracranial artery stenosis may help address this knowledge gap. The current study aimed to investigate the relationship between OSA and intracranial collateral circulation in stroke patients with proximal middle cerebral artery (MCA) stenosis or occlusion (Tables 1 and 2).

**Table 1:** Demographic and clinical characteristics according to OSA status

Variables		OSA		*P*
	without (*n* = 38)	with (*n* = 40)		
Age, mean ± SD	63.1 ± 11.5	59.9 ± 12.7	0.247^a^	
Male gender, n (%)	26 (68.4)	32 (80.0)	0.242^b^	
Body mass index, kg/m2	24.4 ± 3.5	27.3 ± 4.0	0.001^a*^	
Neck circumference, cm	40.2 ± 3.0	41.8 ± 3.4	0.033^a*^	
Waist-to-hip ratio	1.0 ± 0.1	1.0 ± 0.0	0.233^b^	
Hypertension, n (%)	27 (71.1)	30 (75.0)	0.694^b^	
Diabetes, n (%)	14 (36.8)	17 (42.5)	0.610^b^	
Dyslipidaemia, n (%)	9 (23.7)	17 (42.5)	0.078^b^	
Hyperuricaemia, n (%)	8 (21.1)	16 (40.0)	0.070^b^	
Atrial fibrillation, n (%)	2 (5.3)	5 (12.5)	0.432^b^	
Current smoker, n (%)	17 (44.7)	24 (60.0)	0.177^b^	
Alcohol drinking, n (%)	13 (34.2)	8 (20.0)	0.157^b^	
Diastolic blood pressure, mmHg	85.2 ± 12.2	93.9 ± 13.6	0.004^a*^	
Initial NIHSS	2.0 (1.5, 4.0)	4.0 (2.0, 6.0)	0.044^c^	
Homocysteine, µmol/L	12.8 (10.8, 17.6)	15.1 (11.3, 17.8)	0.466^c^	
Haemoglobin A1c, %	6.5 ± 1.4	6.9 ± 1.7	0.238^a^	
Creatinine, µmol/L	69.0 (59.5,79.2)	73.5 (59.7, 85.0)	0.371^c^	
Serum uric acid, mmol/L	308 (255, 348)	365 (313, 424)	0.002^c^	
Total cholesterol, mmol/L	4.0 (3.0, 5.0)	4.6 (3.9, 5.2)	0.069^c^	
LDL-C, mmol/L	2.1 (1.7, 3.1)	2.9 (2.4, 3.2)	0.034^c*^	

*OSA* obstructive sleep apnoea;^a^ independent samples t-test; ^b^ chi-square test; ^c^ Mann‒Whitney U test

**Table 2:** Patient angiographic and sleep study results

Variables	OSA		*P*
	without (*n* = 38)	with (*n* = 40)	
Collateral score ≥ 2, n (%)	25 (65.8)	35 (87.5)	0.023^b*^
Aetiology of stroke, n (%)			0.765^b^
Large artery atherosclerosis	24 (63.2)	23 (57.5)	
Cardioembolism	2 (5.3)	4 (10.0)	
Small vessel occlusion	11 (28.9)	13 (32.5)	
Other or Undetermined	1 (2.6)	0 (0)	
Location of infarction, n (%)			0.999^b^
Anterior circulation	26 (68.4)	27 (67.5)	
Posterior circulation	10 (26.3)	11 (27.5)	
Anterior and posterior	2 (5.3)	2 (5.0)	
AHI,/h	10.2 (7.3, 17)	33.0 (24.0, 43.8)	< 0.001^c*^
ODI,/h	9.0 (6.5, 13.5)	33.5 (22.9, 41.8)	< 0.001^c*^
Minimum SaO_2_, %	80.9 ± 8.4	75.5 ± 11.1	0.018^a^
Mean SaO_2_, %	93.3 ± 2.0	93.2 ± 1.9	0.881^a^
T90, %	1.6 (0.2, 14.9)	4.6 (1.4, 17.2)	0.083^c^
Epworth Sleepiness Scale	6.0 (3.2, 8.0)	9.0 (7.8, 12.0)	< 0.001^c*^

*AHI* Apnoea Hyponea Index, *ODI* oxygen desaturation index, *OSA* obstructive sleep apnoea, *SaO2* arterial oxygen saturation, *T90* percentage of time with SaO2 less than 90; ^a^ independent samples t-test; ^b^ chi-square test; ^c^ Mann‒Whitney U test

^*^p < 0.05

## Methods

### Study design and population

This study enrolled patients with acute ischaemic stroke at the Third Affiliated Hospital of Nanjing Medical University between March 2022 and August 2024. The study was conducted in accordance with the Helsinki Declaration and was approved by the Ethics Committee of the Third Affiliated Hospital of Nanjing Medical University (2024–KY111-01). Owing to the retrospective nature of the study, the requirement for informed consent was waived. The inclusion criteria were as follows: (1) aged ≥ 18 years; (2) diagnosed with ischaemic stroke within 24 h of symptom onset; and (3) confirmed to have unilateral proximal MCA stenosis or occlusion by CT angiography (CTA) within 72 h of stroke onset. The exclusion criteria were as follows: (1) diagnosed with primary haemorrhagic stroke; (2) unstable or life-threatening conditions such as coma, severe heart failure, or persistent oxygen dependency; (3) pregnant; (4) a history of drug or alcohol abuse; (5) unable to follow the study procedures; or (6) predominantly central sleep apnoea (≥ 50% of all respiratory events and central apnoea–hypopnea index [AHI] ≥ 10 events/h) or failed in polygraph recording (without adequate or satisfactory signal recording).

### Assessment of OSA

Patients underwent nocturnal sleep studies during hospitalization from 10 PM to 5 AM with a portable sleep monitoring device (Alice Night one, America). All patients refrained from taking sedatives or hypnotics on the day of testing. The parameters of interest included nasal airflow, chest and abdominal movements, arterial oxygen saturation (by pulse oximetry), and heart rate. Two researchers who were blinded to the clinical characteristics analysed the output data from the portable diagnostic device. They examined all the data and looked for any missing or incorrect judgements. Any discrepancies were resolved by open discussions and consensus. Respiratory events were defined in accordance with the recommendations of the American Academy of Sleep Medicine [20]. Apnoea was defined as complete cessation of airflow for ≥ 10 s (obstructive or central apnoea characterized by the presence of chest and abdominal movement). Hypopnea was defined as a decrease in airflow by > 30% for at least 10 s associated with a decrease in arterial oxygen saturation of at least 4%. The apnoea-hypopnea index (AHI) was calculated as the total number of apnoeas and hypopneas per hour of total recorded time. OSA was defined as an AHI ≥ 15 events/h, whereas non-OSA was defined as an AHI < 15 events/h. The oxygen desaturation index (ODI) was defined as the number of events per hour where oxygen saturation dropped ≥ 4% from baseline. Indicators of nocturnal hypoxemia included the mean 

### CT angiography imaging

**Fig. 1:**
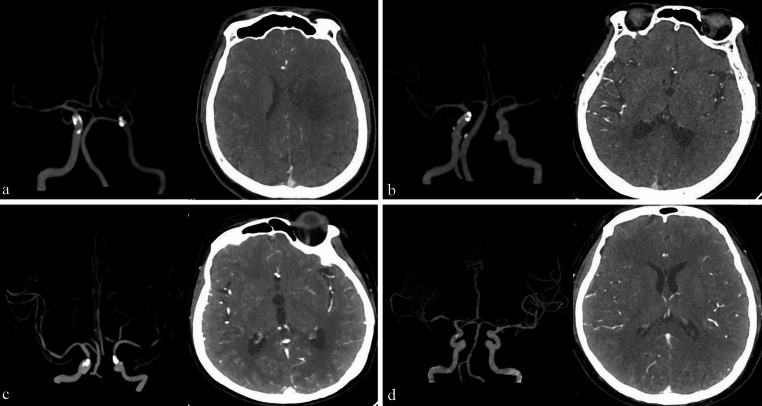
Classification of cerebral collaterals after middle cerebral artery occlusive lesions according to the Tan scoring system. **a**, absence of collateral circulation (Score 0); **b**, leptomeningeal artery collateral circulation ≤ 50% (Score 1); **c**, collateral circulation > 50% but < 100% (Score 2); **d**, complete collateral circulation = 100% (Score 3)

All patients underwent CTA scans, which covered the region from the neck to the calvarium, with a slice thickness of 0.625 mm. Scanning started 5 s after the administration of contrast medium and included 25 consecutive acquisitions over a period of 2 s. All original scan data were subsequently transferred to a dedicated workstation for further analysis. The MCA collateral score was calculated based on the Tan scoring system. The MCA score was used to assess the collateral circulation status of the lesions [21]. Patients were classified into four grades on the basis of the Tan scoring system: absence of collateral circulation (Score 0), leptomeningeal artery collateral circulation ≤ 50% (Score 1), collateral circulation > 50% but < 100% (Score 2), and complete collateral circulation = 100% (Score 3, Fig. 1). Patients were divided into two groups on the basis of these scores: those with good collateral circulation (scores 2 and 3) and those with poor collateral circulation (scores 0 and 1).

### Statistical analysis

Normally distributed continuous variables are presented as the means ± SDs; categorical variables are expressed as frequencies (percentages). Nonnormally distributed variables are reported as medians and interquartile ranges (Q1 - Q3). T tests were used to compare normally distributed continuous variables, and Mann‒Whitney U tests were utilized to compare nonnormally distributed continuous variables. Chi-square tests were employed to compare categorical variables. Wilcoxon rank-sum tests were conducted to assess the relationship between the AHI and the presence of collateral vessels in the MCA. Spearman’s correlation analysis was performed to evaluate the correlation between the AHI and collateral circulation grade. All the statistical analyses were carried out via the Statistical Package for the Social Sciences (SPSS 27.0) and Free Statistics software v1.9.2. A p value of ≤ 0.05 was considered statistically significant.

## Results

**Fig. 2:**
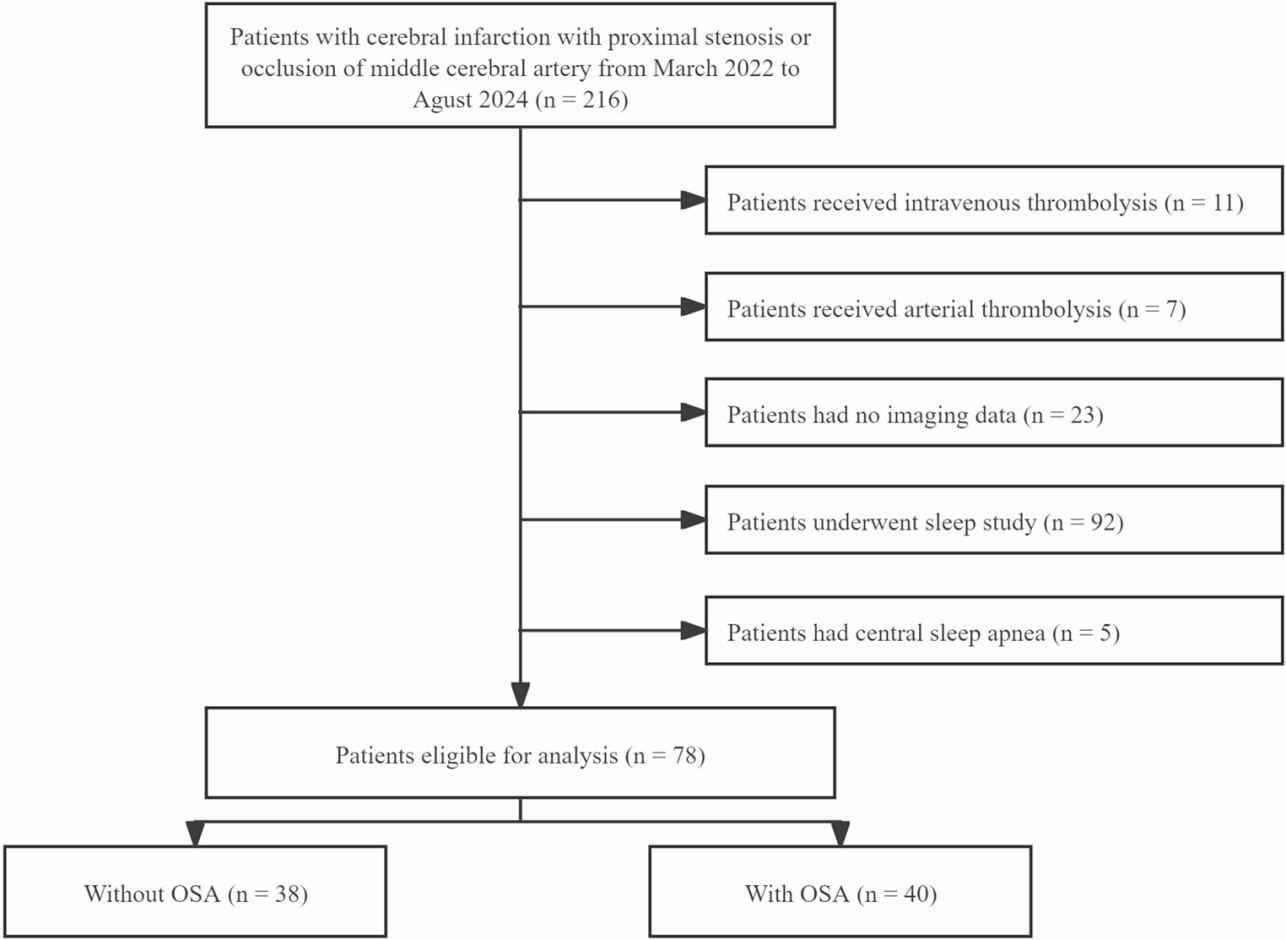
Flow chart for patient enrolment. OSA, obstructive sleep apnoea

During the study period, 216 patients with cerebral infarction were assessed for eligibility, and 138 patients were excluded based on the exclusion criteria (Fig. 2). Among the remaining 78 patients, the proportion of males was 74.3%, and the mean age was 61 ± 12 years. The prevalence of OSA (i.e., AHI ≥ 15/h) was 50.4%. Compared to non-OSA patients, OSA patients presented a greater body mass index (BMI, 27.3 ± 4.0 vs. 24.4 ± 3.5, *P* = 0.001), diastolic blood pressure (93.9 ± 13.6 vs. 85.2 ± 12.2, *P* = 0.004), National Institutes of Health Stroke Scale (NIHSS) score (4.0 vs. 2.0, *P* = 0.044), and waist circumference upon admission (41.8 ± 3.4 vs. 40.2 ± 3.0, *P* = 0.033) (Table 1).

**Fig. 3:**
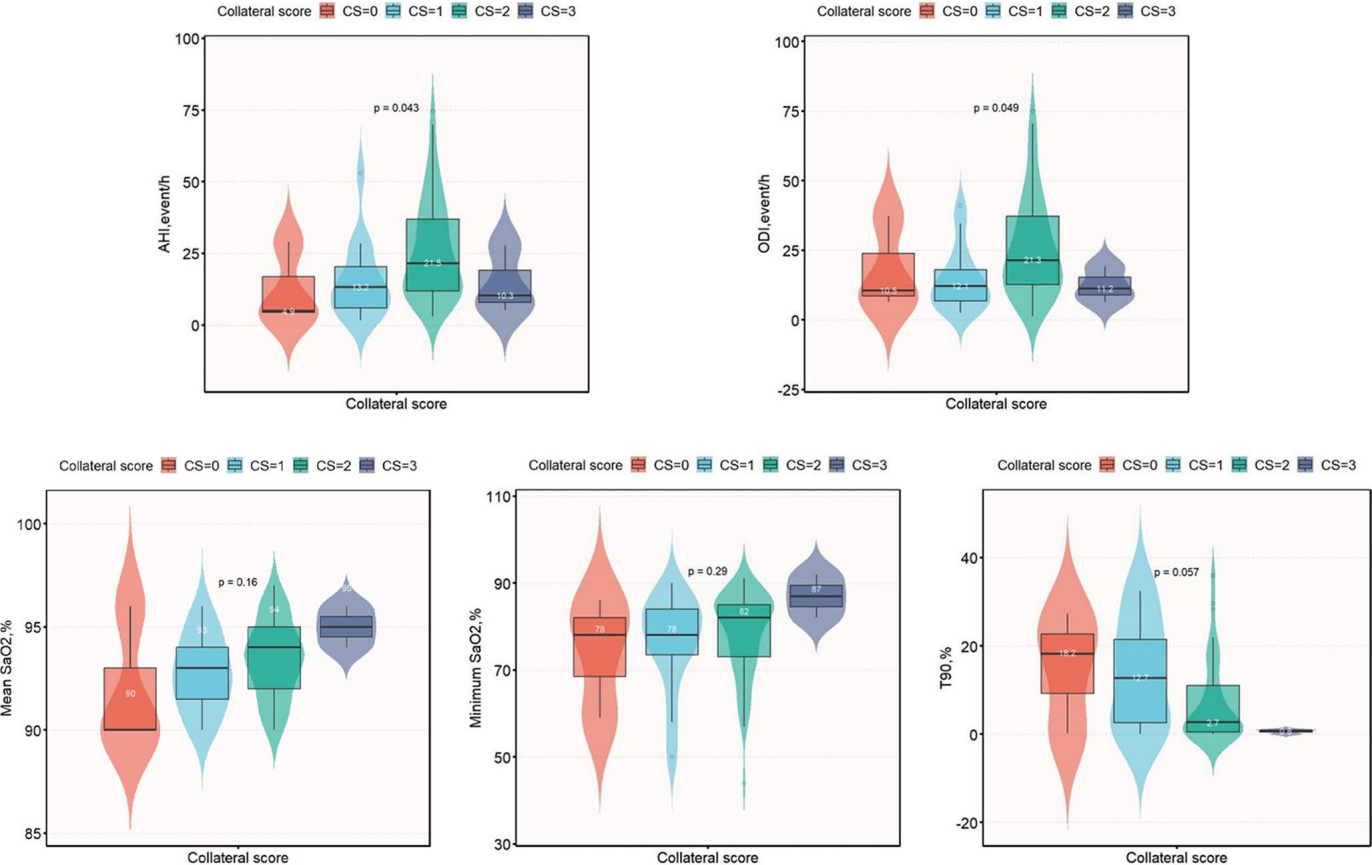
OSA characteristics based on collateral scores. **a**: AHI; **b**: ODI; **c**: Mean SaO2; **d**: Minimum SaO2; and **e**: T90. AHI, apnoea–hypopnea index; ODI, oxygen desaturation index; OSA, obstructive sleep apnoea; SaO2, arterial oxygen saturation; T90, percentage of time with SaO2 less than 90%

There were no significant differences in mean oxygen saturation (SaO2) or the percentage of time with SaO2 less than 90% (T90) between patients with and without OSA. However, OSA patients exhibited significantly better collateral development (collateral score ≥ 2) than patients without OSA (87.5% vs. 65.8%, *P* = 0.023) (Table 2). Moreover, patients with good collateral formation (collateral score ≥ 2) had a significantly greater AHI than did those with poor collateral formation (collateral score < 2, 25.4 ± 17.1 vs. 14.9 ± 13.2, *P* = 0.02). Additionally, increases in the AHI, ODI, and mean SaO2 paralleled increases in collateral scores among all patients except those with a collateral score of 3 (Fig. 3a, b, c). As collateral scores increased, T90 decreased (Fig. 3e). Spearman’s correlation analysis revealed a strong positive correlation between the AHI and collateral scores in patients with proximal MCA stenosis or occlusion due to cerebral infarction (*r* = 0.866, *P* < 0.01).

## Discussion

This study revealed that patients with OSA exhibited superior collateral circulation development compared with those without OSA. Furthermore, the AHI was significantly and positively correlated with the collateral circulation score, while the T90 and average blood oxygen saturation did not exhibit similar associations.

These findings support previous observations regarding the relationship between obstructive sleep apnoea and collateral circulation development. A previous study by Steiner et al. suggested that OSA is associated with the development of coronary collateral circulation [12]. Tao et al. reported that, after adjusting for confounding factors such as clinical characteristics, the presence and severity of OSA significantly the development of coronary collateral circulation in patients with ST-segment elevation myocardial infarction [15]. To date, no studies have established a correlation between OSA and collateral circulation in intracranial vessels. Cerebral collateral circulation refers to alternative or indirect arterial pathways that provide blood flow when the arteries supplying a normal region of brain tissue are occluded. Previous studies have demonstrated that robust collateral circulation can reduce infarct volume and improve clinical outcomes in patients with cerebral infarction [22]. OSA is known to be a strong risk factor for myocardial infarction and stroke. However, recent evidence has suggested that OSA may increase collateral circulation in myocardial infarction patients, thus potentially protecting against myocardial damage [13–15]. Zhang et al. reported a significant negative correlations between the severity of OSA and both infarct volume and neurological recovery among patients with acute cerebral infarction. These findings suggest that OSA may protect the brain before acute ischaemic stroke (AIS) through mechanisms similar to those of ischaemic preconditioning [23]. Via stimulate collateral vessel formation, OSA can result in paroxysmal hypoxia, which may educe neuroprotective responses, a biological process termed as ischemic preconditioning [24]. Hypoxia-inducible factor (HIF), induced during OSA, could increase the level of vascular endothelial growth factor (VEGF) in circulation, and, thereby, induce neovascularization [25, 26]. Animal studies have confirmed that hypoxia state can trigger the formation of new leptomeningeal collaterals even in adults [27]. Additionally, OSA-related hypoxia-reoxygenation could change hemodynamic shear stress, and then facilitate structural remodeling of collateral vessels through luminal expansion and modulation of vascular smooth muscle tone [27, 28]. Rosenzweig et al. reported that patients with OSA presented significantly larger bilateral hippocampi than patients without OSA, thus indicating the formation of new blood vessels [29]. In our study, we found that in patients with cerebral infarction and MCA stenosis, higher AHI values and ODI values were more likely to exhibit better collateral circulation, which is consistent with previous findings on the beneficial effects of OSA on collateral circulation in patients with myocardial infarction. Large-sample clinical trials failed to demonstrated the effectiveness of continuous positive airway pressure (CPAP) therapy for preventing cardiovascular events in patients with OSA. This failure may be explained by the potential role of OSA in promoting collateral circulation [30, 31].

Our study revealed that patients with severe obstructive sleep apnoea presented enhanced collateral development, characterized by higher collateral formation scores (collateral score ≥ 2). Our findings were corroborated by Spearman’s correlation analysis, which revealed a positive correlation between the AHI and the collateral grade of the MCA. The results indicated that as the collateral grade increased, the AHI, ODI, and mean SaO2 level also increased, whereas T90 decreased. These observations suggest an alternative mechanism whereby chronic severe hypoxemia due to OSA might paradoxically inhibit collateral circulation development, thereby impacting the condition and prognosis of patients with cerebral infarction. While OSA may have protective effects on collateral formation during acute cerebral infarction, numerous studies have indicated that it is also linked to negative outcomes that should not be ignored. Various genetic, lifestyle, and treatment factors likely contribute to these observed differences, making OSA a context-dependent risk factor. Festic et al. introduced the term “dual effect” to describe how OSA can produce both pathological cardiovascular effects and improved outcomes in some patients following ischaemic events.

This study has several limitatins. First, the retrospective nature of our study and its small sample size may restrict the generalizability of our results. Second, this study did not systematically assess potential confounding factors such as vascular disease history, medication use and physical activity levels, which may influence the interpretation of the findings. Future studies are warranted to confirming these findings.Patients were diagnosed with sleep-disordered breathing (SDB) via overnight polysomnography instead of full polysomnography, which is considered the gold standard. However, portable respiratory devices have been widely validated for assessing SDB in hospitalized patients when polysomnography is not feasible. The impact of collateral circulation on future events in patients with OSA combined with cerebral infarction requires further investigation.

In summary, the AHI may facilitate the development of collateral circulation in patients with acute cerebral infarction. However, severe and prolonged hypoxia associated with OSA could inhibit neovascularization, thereby negatively impacting collateral circulation. Future studies should involve larger prospective designs to clarify how OSA influences the development of collateral circulation in patients with cerebral infarction.

## Data Availability

The datasets generated during and/or analyzed during the current study are available from the corresponding author on reasonable request.
